# Supplementary material for: Porcine Circovirus Modulates Swine Influenza Virus Replication in Pig Tracheal Epithelial Cells and Porcine Alveolar Macrophages
Source: Viruses. 2023 May 20;15(5):1207. doi: 10.3390/v15051207 (PMC10222781; doi:10.3390/v15051207)
Supplement: Supplementary file 1 [file viruses-15-01207-s001.zip › Supplemental Figures.pdf]

Supplemental figures

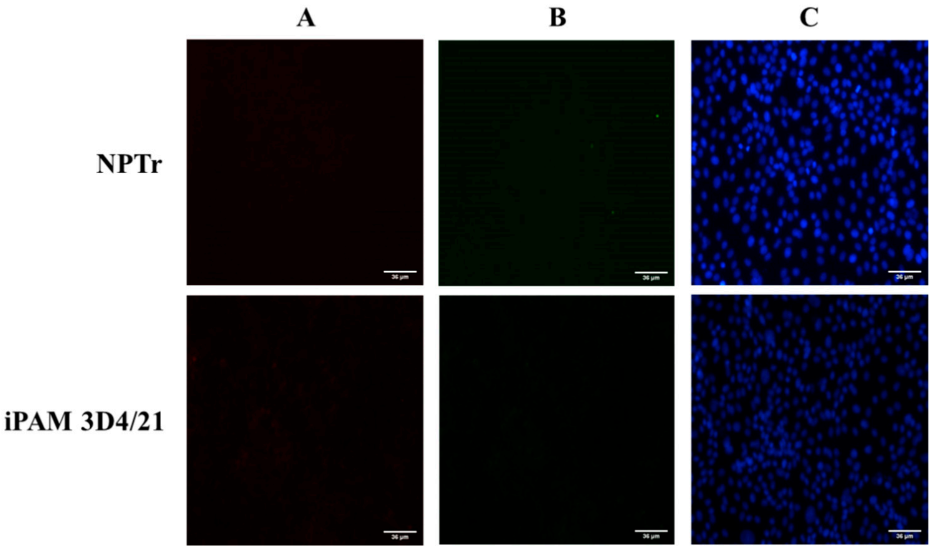

Supplemental Figure S1. Immunofluorescence assay performed on NPTr and iPAM 3D4/21 mock-infected cells. A) The NPTr and iPAM 3D4/21 mock- infected cells were incubated with the polyclonal PCV2 porcine antiserum, followed by and incubation step with a goat anti-swine rhodamine conjugated secondary antibody (Jackson ImmunoResearch, PA, USA), B) The NPTr and iPAM 3D4/21 mock-infected cells were incubated with the monoclonal mouse anti-NP SwIV primary antibody (Bio-Rad), followed by and incubation step with a goat anti-mouse FITC conjugated secondary antibody (Invitrogen, CA, USA), C) Nuclear staining with DAPI is shown in blue. The immunofluorescence assay was performed as described in the materials and methods section.

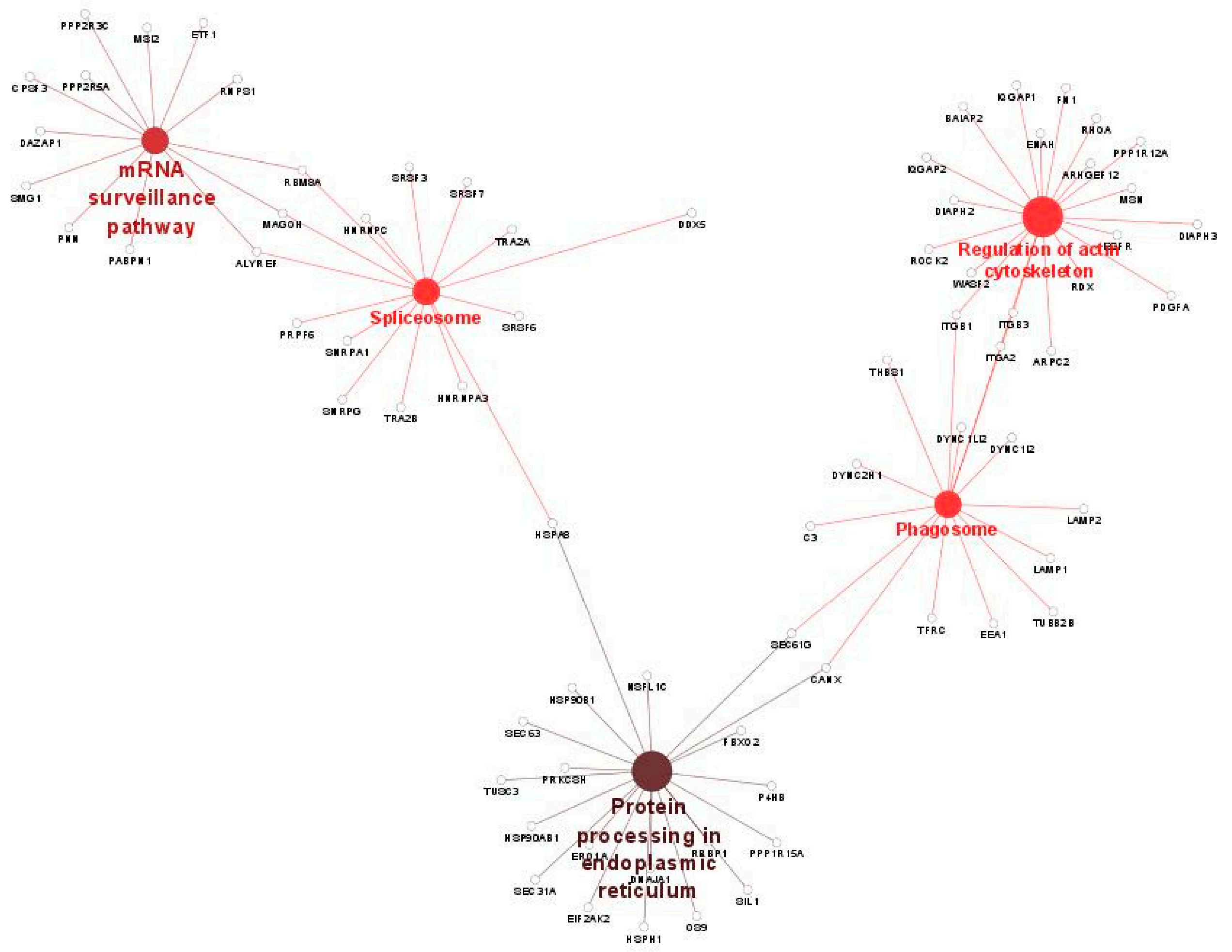

Supplemental Figure S2. Protein-protein interaction networks and associated cellular pathways on PCV2b/swH1N1 co-infected NPTr cells.

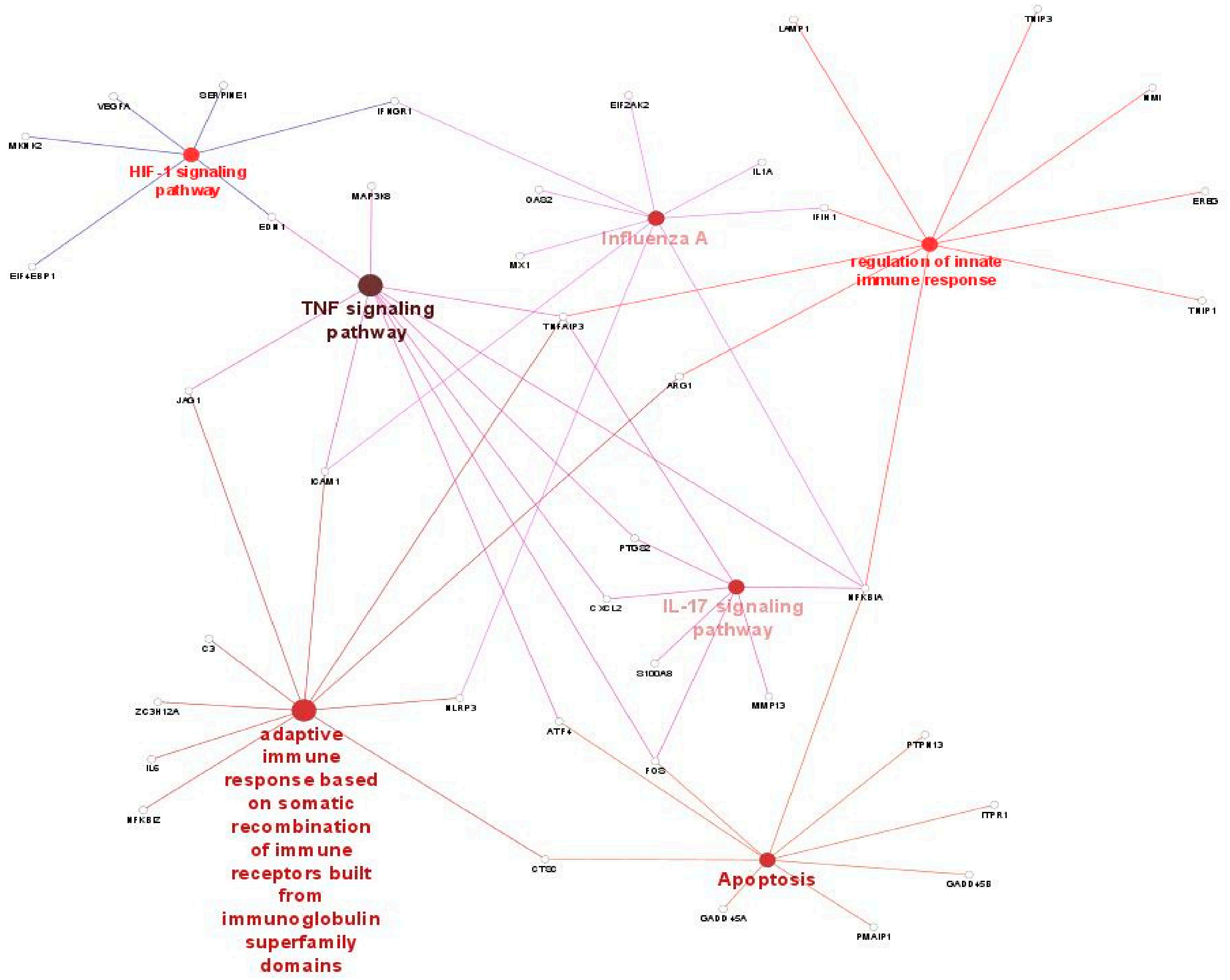

Supplemental Figure S3. Protein-protein interaction networks and associated cellular pathways on PCV2b/swH1N1 co-infected iPAM 3D4/21 cells.
