# Supplementary material for: Porcine Circovirus Modulates Swine Influenza Virus Replication in Pig Tracheal Epithelial Cells and Porcine Alveolar Macrophages
Source: Viruses. 2023 May 20;15(5):1207. doi: 10.3390/v15051207 (PMC10222781; doi:10.3390/v15051207)
Supplement: Supplementary file 1 [file viruses-15-01207-s001.zip › Supplemental Table S1. List of primers .pdf]

Supplemental Table S1. List of primers and probe used for PCV2b quantification and primers used in RT-qPCR for cytokines mRNA quantification in NPTr and iPAM 3D4/21 cells

Target	Primers and probes 5'- 3'	References
PCV2 (ORF1)	F: GGC CAC CTG GGT GTG GTA AA<br>R: CCC ACC ACT TGT TTC TAG GTG GTT<br>Probe 6-FAM-TTT GCA GAC CCG GAA ACC ACA TAC TGG A-BHQ-1)	[11]
IL-6	F: ACTCCCTCTCCACAAGCGCCTT<br>R: TGGCATCTTCTTCCAGGCGTCCC	[53]
IL-8	F: TGTGAGGCTGCAGTTCTGGCAAG<br>R: GGGTGGAAAGGTGTGGAATGCGT	
IL-10	F: GCTGCGGCGCTGTCATCAATT<br>R: ACCCATGGCTTTGTAGACACCCC	
IFN-α	F: ACTCCATCCTGGCTGTGAGGAAAT<br>R: TCTGTCTTGCAGGTTTGTGGAGGA	[54]
IFN-β	F: CTCTCCTGATGTGTTTCTCC<br>R: GTTCATCCTATCTTCGAGGC	
IFN- γ	F: GAGCCAAATTGTCTCCTTCTAC<br>R: CGAAGTCATTCAGTTTCCCAG	
B2M	F: GTGCTATCTCCACGTTTGAG<br>R: GCTTCGAGTGCAAGAGATTG	
B-actin	F: ACCACTGGCATTGTCATGGACTCT<br>R: ATCTTCATGAGGTAGTCGGTCAGG	
PPIA	F: TGCAGACAAAGTTCCAAAGACAG<br>R: GCCACCAGTGCCATTATGG	
