# Supplementary material for: Porcine Circovirus Modulates Swine Influenza Virus Replication in Pig Tracheal Epithelial Cells and Porcine Alveolar Macrophages
Source: Viruses. 2023 May 20;15(5):1207. doi: 10.3390/v15051207 (PMC10222781; doi:10.3390/v15051207)
Supplement: Supplementary file 1 [file viruses-15-01207-s001.zip › Supplemental Table S2. Up-rgulated genes in NPTr cells.pdf]

Supplemental Table S2. Up-regulated genes in NPTr cells

PCV2b/H1N1		PCV2b		H1N1	
Gene ID	Fold change	Gene ID	Fold change	Gene ID	Fold change
PRDM5	250,86	CCK	161,38	SERPINH1	12,97
CHRNG	99,44	PRDM5	101,99	SDCBP	10,46
CXCL8	96,60	TGFBR3	20,66	gene:ENSSSCG00000001917	9,15
CXCL2	65,63	gene:ENSSSCG00000046628	18,52	PSMB1	7,91
CCK	62,28	CXCL2	17,51	gene:ENSSSCG00000012119	6,77
VAT1L	53,32	PPP1R14C	17,45	NDRG1	6,51
gene:ENSSSCG00000046628	31,74	DNAJB6	11,12	TACSTD2	5,61
LTF	29,40	PSPH	11,12	SLC25A3	5,27
PPP1R14C	24,66	SNRPA1	10,20	CCN1	5,21
PROK2	22,62	PPP1R10	9,29	FADS2	5,03
CCN2	22,03	TNIP3	9,28	MRFAP1	4,87
PSPH	21,36	EDN1	9,02	TAX1BP3	4,35
IQUB	19,57	DDX52	8,64	SLC50A1	4,30
ALYREF	19,44	ZNF646	8,51	ERBB3	4,20
NPM3	18,04	CSKMT	7,95	MGST3	4,04
IL1A	17,54	gene:ENSSSCG00000047320	7,22	IGFBP2	3,86
SRSF7	16,52	ZNF131	6,99	gene:ENSSSCG00000035570	3,86
MRPL15	15,61	DUSP4	6,57	UNC5B	3,83
PLAU	14,81	SGK1	6,53	ITM2C	3,83
CDC42SE1	14,20	TRIB3	6,53	SKP1	3,73
SEC61G	14,12	RCSD1	6,51	FHL2	3,72
SDCBP	13,51	ZNF12	5,88	CLDN1	3,69
TNIP3	13,13	NCLN	5,79	LGALS3	3,55
RCSD1	13,09	MAGOH	5,79	PTP4A1	3,54
P2RY1	12,59	SRSF6	5,75	ATP5PO	3,45
CSKMT	12,50	ZC3H12A	5,74	PRKAR1A	3,45
CCN1	12,21	ODC1	5,49	ESD	3,33
SGK1	11,82	UBTF	5,45	PICALM	3,32
ZNF646	11,71	CUTC	5,45	FTL	3,28
NSFL1C	11,71	SLC38A2	5,18	NDUFC2	3,26
gene:ENSSSCG00000012119	10,95	GIPC1	5,05	PFKFB3	3,22
CHCHD1	10,89	gene:ENSSSCG00000033786	4,74	HINT1	3,17
ART4	10,64	HSPH1	4,50	PGK1	3,11
SNRPA1	10,09	PTGS2	4,42	MMP13	3,09
GPATCH4	9,91	MED9	4,38	PPP1CB	3,08

DDX52	9,86	AMD1	4,32	NIBAN2	3,07
ANKRD49	9,28	ANKRD1	4,27	DDR1	3,05
gene:ENSSSCG00000051136	9,02	SAFB2	4,26	UBB	2,81
ODC1	8,82	PPP1R15A	4,25	WASL	2,80
TRIB3	8,79	ETS2	4,21	HPS5	2,80
NUDT16	8,73	gene:ENSSSCG00000016733	4,11	RPS29	2,80
MRPS18C	8,68	PCLAF	3,90	gene:ENSSSCG00000017509	2,76
ZNF639	8,68	TNFSF9	3,75	PSAP	2,60
gene:ENSSSCG00000044847	8,36	AAK1	3,75	ITM2B	2,58
RND1	8,35	MAFF	3,74	gene:ENSSSCG00000007743	2,57
CCNB1IP1	8,22	SYS1	3,73	DDIT4	2,57
DUSP4	8,21	WDR74	3,66	HES1	2,54
SLC38A2	8,07	BRIX1	3,66	MMP7	2,46
H2AFZ	7,86	NOP56	3,66	MAL2	2,41
MAGOH	7,85	HSPA8	3,65	gene:ENSSSCG00000014071	2,35
DUSP5	7,64	ELOA	3,64	gene:ENSSSCG00000004489	2,33
MMP13	7,63	NOP58	3,63	NFKBIA	2,29
OSTC	7,46	TNFAIP3	3,62	CST3	2,27
SEMA7A	7,45	PEDS1	3,59	gene:ENSSSCG00000005453	2,22
TGFBR3	7,40	gene:ENSSSCG00000022322	3,52	gene:ENSSSCG00000033697	2,22
PSMB1	7,36	MUC13	3,52	RAC1	2,15
PPP1R15A	7,06	PEA15	3,46	RPLP0	2,12
NOP58	6,97	ZNF207	3,44	PMEPA1	2,09
THOC6	6,81	GATAD2A	3,41	RPL23	2,04
gene:ENSSSCG00000030300	6,70	TCF7L2	3,37	RPS3A	2,00
UGCG	6,69	PDRG1	3,24	RPS25	2,00
CTSC	6,65	GID4	3,23	ALDH1A1	2,00
DYNLT1	6,59	STK4	3,13	RPS7	1,97
WDR74	6,41	RNF44	3,12	RPL34	1,95
PNO1	6,39	gene:ENSSSCG00000034711	3,11	EIF3E	1,95
MCL1	6,38	gene:ENSSSCG00000011803	3,10	TPT1	1,91
PTGS2	6,38	MKNK2	3,10	RPL27	1,85
RELA	6,21	RNPS1	3,07	LAPTM4A	1,83
CDC42EP3	6,19	CTR9	3,06	ENO1	1,82
SRSF6	6,17	CTSC	3,03	SPP1	1,81
NLRP3	6,16	SAT1	2,94	ZFP36L2	1,80
EDN1	6,12	SRXN1	2,89		
NOP56	6,09	RBM8A	2,89		
SAFB2	6,08	ZNFX1	2,87		
AMD1	6,07	POLR3D	2,79		
KRT10	6,03	DDX23	2,79		

PCLAF	5,91	EIF3J	2,78		
gene:ENSSSCG00000050998	5,86	ING3	2,75		
MLLT11	5,86	PSMD7	2,73		
HILPDA	5,84	R3HDM4	2,65		
gene:ENSSSCG00000032059	5,82	ZFP36L1	2,62		
ZNF131	5,76	FUNDC2	2,61		
AKAP8	5,76	DHX38	2,60		
MRPL43	5,76	TXNIP	2,58		
PNP	5,74	ETV3	2,58		
TIMM8B	5,59	SQSTM1	2,48		
TMEM87B	5,51	DDX3X	2,42		
CLDN1	5,43	IDI1	2,35		
TACSTD2	5,36	HBEGF	2,34		
FERMT1	5,31	gene:ENSSSCG00000009211	2,33		
AAK1	5,30	IER3	2,29		
NFKBIA	5,27	TM4SF1	2,29		
gene:ENSSSCG00000029160	5,22	MT1A	2,21		
RBP4	5,19	PSMD8	2,17		
EIF3J	5,16	FNDC7	2,17		
TRAPPC8	5,15	UBE2Q1	2,08		
TFRC	5,15	CD24	1,95		
CUTC	5,15	COX1	1,89		
TNFSF9	5,14				
HSPA8	5,13				
gene:ENSSSCG00000009211	5,11				
PRNP	5,04				
PEDS1	5,02				
S100A8	4,83				
gene:ENSSSCG00000047320	4,81				
GRPEL2	4,76				
gene:ENSSSCG00000017626	4,76				
RPL7L1	4,73				
RAN	4,67				
CYCS	4,64				
ELOA	4,59				
MED9	4,51				
POLR2G	4,49				
SMCR8	4,48				
gene:ENSSSCG00000011803	4,47				
gene:ENSSSCG00000014031	4,43				
SNRPG	4,42				

TRA2A	4,39				
gene:ENSSSCG00000005478	4,30				
gene:ENSSSCG00000033786	4,21				
TXNIP	4,20				
SRSF3	4,15				
gene:ENSSSCG00000022322	4,12				
ERBB3	4,12				
TRA2B	4,11				
RNF220	4,10				
ZC3H12A	4,07				
RNF19B	4,03				
NDUFB1	4,03				
MPHOSPH10	4,03				
HSPH1	4,01				
HINT1	4,00				
MRFAP1	3,98				
OSER1	3,89				
RNF126	3,87				
TRMT13	3,81				
NADK	3,79				
LGALS3	3,76				
VMP1	3,68				
ITPRID2	3,67				
PSMD12	3,65				
SREK1IP1	3,54				
EXOSC1	3,49				
PPP1R14B	3,49				
PTP4A1	3,46				
RIT1	3,41				
DKC1	3,36				
gene:ENSSSCG00000039071	3,33				
RNPS1	3,32				
SNRPB	3,31				
EREG	3,28				
HNRNPA0	3,27				
MAP2K3	3,27				
gene:ENSSSCG00000040889	3,26				
RBM8A	3,25				
SLIRP	3,23				
DHRS9	3,23				
PSMD7	3,20				

BRD2	3,20				
PABPN1	3,19				
TM4SF1	3,18				
MAP7D1	3,14				
HNRNPC	3,13				
PBDC1	3,07				
ITM2B	3,04				
HNRNPA3	3,04				
YTHDF2	3,04				
DDX3X	3,00				
KRR1	2,96				
DENND2C	2,95				
IFNAR1	2,94				
DDX21	2,87				
gene:ENSSSCG00000038359	2,84				
DDX5	2,84				
gene:ENSSSCG00000003079	2,83				
SYS1	2,78				
FHAD1	2,74				
DNAJA1	2,73				
BRIX1	2,72				
H2AZ2	2,72				
SMIM7	2,71				
gene:ENSSSCG00000005453	2,69				
USP1	2,69				
PTBP1	2,68				
gene:ENSSSCG00000034572	2,68				
THBS1	2,62				
DDX27	2,61				
MCM4	2,58				
ETS2	2,54				
ZFP36L1	2,50				
ZNFX1	2,49				
COX7A2	2,49				
RTF1	2,48				
POLR2K	2,46				
PEA15	2,41				
COX1	2,39				
EMP1	2,39				
ETF1	2,38				
MT1A	2,37				

FAM133B	2,34				
BCLAF1	2,34				
POMP	2,31				
MRPL57	2,30				
MKNK2	2,29				
TNFAIP3	2,28				
PSMD8	2,27				
CCT5	2,22				
RSL24D1	2,16				
PRDX1	2,14				
PNN	2,08				
ANXA1	2,07				
gene:ENSSSCG00000000377	2,02				
ATP6	1,88				

Fold change of gene expression in infected cells comparing to control (mock-infected cells).
