# Supplementary material for: Porcine Circovirus Modulates Swine Influenza Virus Replication in Pig Tracheal Epithelial Cells and Porcine Alveolar Macrophages
Source: Viruses. 2023 May 20;15(5):1207. doi: 10.3390/v15051207 (PMC10222781; doi:10.3390/v15051207)
Supplement: Supplementary file 1 [file viruses-15-01207-s001.zip › Supplemental Table S3. Down-rgulated genes in NPTr cells.pdf]

Supplemental Table S3. Down-regulated genes in NPTr cells

PCV2b/H1N1		PCV2b		H1N1	
Gene ID	Fold change	Gene ID	Fold change	Gene ID	Fold change
DIAPH2	-18,95	YPEL2	-21,80	gene:ENSSSCG00000043033	-8,37
SWI5	-18,26	PAPSS1	-18,98	CCDC47	-4,61
CHD6	-14,14	gene:ENSSSCG00000015491	-16,28	ING3	-4,11
FAM114A1	-13,86	ZBTB37	-14,77	UBAP2L	-4,06
PPP1R9A	-13,08	SMARCA2	-13,94	gene:ENSSSCG00000002722	-3,06
YPEL2	-12,59	SFT2D2	-13,77	gene:ENSSSCG00000015337	-2,90
gene:ENSSSCG00000035037	-12,35	NXN	-12,01	EMILIN2	-2,86
RCAN2	-12,06	SLC5A3	-11,40	MICU2	-2,84
SLC5A3	-11,95	SYPL1	-9,79	PSMC3	-2,74
PRKCI	-11,72	PTPRG	-9,59	RBM25	-2,74
SGSM1	-11,45	NET1	-9,39	LAMC1	-2,68
NBAS	-11,43	OS9	-9,14	LAP3	-2,43
GUSB	-10,96	SYNE2	-8,88	HK1	-2,29
SCEL	-10,85	MED4	-8,73	gene:ENSSSCG00000035209	-2,27
PPP2R5A	-10,69	CADM1	-8,66	HSPD1	-2,26
LAMP2	-10,56	EMB	-8,48	EEA1	-2,25
VPS13C	-10,30	HLTF	-8,32	gene:ENSSSCG00000042436	-2,20
KANK2	-9,75	gene:ENSSSCG00000035037	-8,24	gene:ENSSSCG00000022176	-2,19
MTURN	-9,54	CMTM4	-8,20	CCN3	-2,07
TNIK	-9,40	ATP11C	-8,09	gene:ENSSSCG00000043470	-2,07
UACA	-9,32	RCAN2	-7,97	FNBP1L	-2,01
LIMCH1	-9,26	ITGB4	-7,92	gene:ENSSSCG00000036350	-2,01
CADM1	-9,22	SEC31A	-7,92	SMARCC1	-1,90
R3HDM2	-9,15	MYO6	-7,43	PTGES	-1,85
NXN	-8,66	SIL1	-7,43	ITGA2	-1,71
FNBP1L	-8,66	DLG1	-7,20	HSP90AB1	-1,68
PDK3	-8,53	FARP1	-7,09	SYVN1	-1,66
ATP11C	-8,52	SMPDL3B	-7,05		
SGMS2	-8,41	FAM114A1	-7,02		
CGNL1	-8,39	FAM162A	-6,96		
SEC31A	-8,31	gene:ENSSSCG00000040337	-6,91		
GOLM1	-8,17	ATRX	-6,87		
GPC1	-8,13	ARHGAP5	-6,69		
gene:ENSSSCG00000004511	-7,96	DPCD	-6,68		
NF1	-7,79	PMEPA1	-6,59		

SIL1	-7,79	TCEA3	-6,59		
USO1	-7,72	ASH1L	-6,58		
OXR1	-7,67	UBXN1	-6,35		
FCHSD2	-7,66	STRBP	-6,35		
SACM1L	-7,66	SGSM1	-6,18		
SMG1	-7,63	USO1	-6,17		
COPB1	-7,60	PTPRF	-6,16		
ATRX	-7,57	TSPO	-6,07		
SGPP2	-7,57	MTSS1	-6,06		
PPP1R21	-7,54	IQGAP2	-6,05		
gene:ENSSSCG00000012074	-7,54	PRKCI	-5,96		
GPBP1	-7,37	MYOF	-5,91		
gene:ENSSSCG00000026996	-7,34	BIRC6	-5,87		
PPME1	-7,32	MRPL37	-5,83		
SYNE2	-7,32	NBAS	-5,81		
SLC39A10	-7,24	ARHGAP18	-5,81		
EXOC1	-7,17	C3	-5,81		
SLC44A2	-7,13	CEP290	-5,79		
DPP4	-7,09	VPS13C	-5,79		
DCTN2	-7,00	gene:ENSSSCG00000004387	-5,68		
BBX	-6,97	PDK3	-5,67		
PATJ	-6,94	CLSTN1	-5,66		
SPTBN1	-6,89	UTRN	-5,61		
gene:ENSSSCG00000009501	-6,82	ARFIP1	-5,52		
COL5A1	-6,82	PPP1R21	-5,51		
TNRC6B	-6,80	XPO1	-5,48		
CLCN5	-6,74	gene:ENSSSCG00000040990	-5,47		
EIF2AK2	-6,74	CYP20A1	-5,42		
APPL1	-6,71	gene:ENSSSCG00000022032	-5,39		
PCMTD1	-6,70	CPNE3	-5,30		
PALLD	-6,70	KIF20B	-5,25		
STRBP	-6,68	TMEM30A	-5,25		
DTNA	-6,66	ASPH	-5,02		
ATP2B4	-6,65	NPNT	-4,89		
ACBD3	-6,63	DSP	-4,86		
MICU2	-6,58	KANK2	-4,85		
CUEDC1	-6,58	AGRN	-4,83		
ATXN10	-6,57	IGFBP7	-4,81		
MIPEP	-6,54	LIMCH1	-4,71		
gene:ENSSSCG00000015491	-6,54	NDUFS4	-4,63		
ROCK2	-6,44	gene:ENSSSCG00000017509	-4,61		

TGFB1	-6,44	USP8	-4,58		
KCNK12	-6,15	TM9SF4	-4,55		
ROBO1	-6,15	TRMT10A	-4,55		
BICC1	-6,14	MEST	-4,52		
ILRUN	-6,14	SCEL	-4,46		
NAB1	-6,10	gene:ENSSSCG00000012074	-4,46		
ACBD6	-6,09	FNDC3A	-4,44		
NRDC	-6,09	COX7A2L	-4,41		
ZNF292	-6,09	ACAP2	-4,35		
TMEM52	-6,06	gene:ENSSSCG00000026996	-4,35		
RHOA	-5,99	USP14	-4,34		
EGFR	-5,94	CXCL14	-4,27		
DIAPH3	-5,94	F3	-4,18		
C1orf21	-5,92	gene:ENSSSCG00000049831	-4,15		
ARFIP1	-5,79	PRRG1	-4,12		
ASPM	-5,76	CALD1	-4,05		
IQGAP2	-5,75	ITGAV	-4,03		
DYNC2H1	-5,73	ECHS1	-4,03		
SORT1	-5,71	ERC1	-3,99		
ASH1L	-5,67	THRAP3	-3,98		
CFDP1	-5,65	LAMP2	-3,98		
gene:ENSSSCG00000020686	-5,63	ZNF292	-3,95		
JAK1	-5,61	ARHGEF12	-3,95		
LOXL2	-5,55	VIRMA	-3,94		
CPSF3	-5,55	BBX	-3,93		
SFT2D2	-5,53	TPM1	-3,93		
RAI14	-5,53	TEAD1	-3,92		
KIF20B	-5,52	LARS1	-3,90		
UBR4	-5,52	SEMA3C	-3,88		
MYO6	-5,44	CNOT1	-3,85		
CCN3	-5,42	gene:ENSSSCG00000040056	-3,76		
LARS1	-5,41	DCBLD2	-3,75		
ARCN1	-5,38	SGPP2	-3,71		
KRT18	-5,34	TM9SF2	-3,71		
CALD1	-5,34	CDH6	-3,66		
HLA-DRA	-5,33	UBXN4	-3,65		
gene:ENSSSCG00000020872	-5,32	PDK1	-3,65		
UTRN	-5,13	ROCK2	-3,64		
IFT57	-5,13	GOLM1	-3,60		
XPO7	-5,11	CPSF3	-3,57		
PAWR	-5,08	ROBO1	-3,57		

ITPR2	-5,03	IDH2	-3,54		
HK1	-4,95	MAP1B	-3,54		
C3	-4,94	ARFGAP3	-3,52		
RSRC1	-4,89	ADGRG1	-3,52		
NDUFS4	-4,86	AP2B1	-3,50		
DNMT1	-4,85	IGF2BP2	-3,49		
CNOT1	-4,81	VGLL4	-3,49		
ERBIN	-4,81	ACBD3	-3,49		
LAMB3	-4,79	MFF	-3,48		
gene:ENSSSCG00000042436	-4,71	P4HA1	-3,46		
BIRC6	-4,70	LAMB3	-3,44		
gene:ENSSSCG00000001455	-4,66	STAG2	-3,44		
SMARCA2	-4,66	GM2A	-3,43		
KTN1	-4,65	FN1	-3,41		
NAGA	-4,61	SH3GLB1	-3,39		
CALCOCO2	-4,58	LOXL2	-3,38		
gene:ENSSSCG00000015011	-4,57	CCNI	-3,38		
USP14	-4,56	CTNNA1	-3,37		
NES	-4,56	VPS36	-3,36		
ZMYND11	-4,55	DIAPH2	-3,34		
MYOF	-4,53	DYNC1LI2	-3,33		
NUCB1	-4,52	COL5A1	-3,32		
DCDC2	-4,49	PCM1	-3,31		
PPFIBP1	-4,49	CAMK2D	-3,30		
gene:ENSSSCG00000007322	-4,49	JAK1	-3,29		
CCDC80	-4,45	MTURN	-3,29		
IQGAP1	-4,44	gene:ENSSSCG00000034871	-3,27		
PRPF6	-4,44	gene:ENSSSCG00000028092	-3,25		
ARL13B	-4,39	SLC22A18	-3,25		
SEMA3C	-4,39	TPP1	-3,23		
LAMC1	-4,37	RAB10	-3,22		
AP3B1	-4,34	SKIL	-3,21		
PDS5B	-4,33	DTNA	-3,21		
PM20D2	-4,31	PPME1	-3,21		
PTPRG	-4,31	EGFR	-3,21		
FAM13A	-4,31	LAD1	-3,20		
CLTC	-4,27	CETN3	-3,18		
PLOD1	-4,26	SORT1	-3,17		
IDH2	-4,24	SUPT3H	-3,16		
KRT8	-4,23	LAMC2	-3,14		
ARFGAP3	-4,22	LRPAP1	-3,14		

PDK1	-4,19	ENO1	-3,11		
ERC1	-4,19	CD2AP	-3,11		
PFKP	-4,19	TUBB2B	-3,10		
gene:ENSSSCG00000003047	-4,19	KRT14	-3,09		
WDR1	-4,16	NF1	-3,08		
AGRN	-4,16	IQGAP1	-3,07		
FBXO2	-4,15	CD44	-3,07		
ARHGEF12	-4,15	BICC1	-3,07		
STAG2	-4,13	SYT11	-3,05		
RALA	-4,10	ATP6V1C1	-3,03		
EIF2A	-4,08	RAB6A	-3,03		
WASF2	-4,06	FAM13A	-3,01		
STRN	-4,05	CCDC88A	-3,01		
CD2AP	-4,04	BAZ1B	-3,01		
ASCC3	-4,03	BNIP3L	-3,00		
NSMCE1	-4,02	COPB1	-2,99		
TPM1	-4,00	gene:ENSSSCG00000009501	-2,99		
ACAP2	-3,95	DHX29	-2,98		
RIF1	-3,94	ATP2B4	-2,97		
gene:ENSSSCG00000006156	-3,94	APLP2	-2,95		
HLTF	-3,92	DEPDC1B	-2,94		
DLG1	-3,89	RBM26	-2,94		
DDB1	-3,86	ATP1A1	-2,94		
SMPDL3B	-3,86	CALCOCO2	-2,93		
IGF2BP2	-3,85	OPTN	-2,93		
RNF13	-3,84	KIF2A	-2,91		
gene:ENSSSCG00000004387	-3,84	PDGFA	-2,90		
MSI2	-3,81	UACA	-2,87		
HSPG2	-3,79	SMARCC1	-2,86		
OS9	-3,79	ME1	-2,85		
DAZAP1	-3,78	DCTN2	-2,85		
gene:ENSSSCG00000040990	-3,78	EXOC1	-2,84		
ZNF609	-3,77	MICU2	-2,83		
GOLGB1	-3,75	gene:ENSSSCG00000042436	-2,82		
LAD1	-3,74	KRT18	-2,80		
EMILIN2	-3,73	KRT8	-2,79		
EEA1	-3,71	CRK	-2,78		
GOLGA4	-3,70	EIF4G3	-2,77		
FNDC3A	-3,69	ENAH	-2,77		
SLC22A18	-3,64	FNBP1L	-2,76		
TRMT10A	-3,63	PPFIBP1	-2,75		

TRIP11	-3,63	ITGB1	-2,74		
GFPT1	-3,63	ARHGAP29	-2,71		
SRBD1	-3,63	GNB1	-2,71		
UGP2	-3,63	PPIG	-2,71		
ZC3HAV1	-3,63	KTN1	-2,70		
ITGB1	-3,62	RRBP1	-2,69		
GRN	-3,61	CTSH	-2,68		
ENAH	-3,57	gene:ENSSSCG00000008183	-2,68		
APLP2	-3,56	PTBP3	-2,68		
RRBP1	-3,55	CALM3	-2,66		
LRPAP1	-3,54	PRC1	-2,66		
CEP290	-3,54	FLNB	-2,65		
SMARCC1	-3,53	ZRANB2	-2,63		
BHLHE41	-3,52	ITPR2	-2,62		
MAP1LC3A	-3,52	DDX1	-2,61		
POLE4	-3,49	NPEPPS	-2,60		
SKA2	-3,49	ZNF395	-2,57		
SYT11	-3,49	LMO7	-2,56		
CAMK2D	-3,49	KLF10	-2,55		
VEGFA	-3,48	GOLGB1	-2,54		
gene:ENSSSCG00000014909	-3,47	ST13	-2,53		
SUMF1	-3,47	CDC5L	-2,52		
AIRE	-3,47	SLK	-2,48		
gene:ENSSSCG00000001924	-3,47	gene:ENSSSCG00000034853	-2,48		
PCM1	-3,47	SUMF1	-2,48		
NPNT	-3,46	CENPF	-2,47		
PRC1	-3,46	CBX5	-2,46		
ME1	-3,46	ERO1A	-2,44		
FUOM	-3,44	gene:ENSSSCG00000040827	-2,44		
UBXN1	-3,44	P4HB	-2,43		
ITGA2	-3,43	TGFB1	-2,42		
ASPH	-3,41	HSPG2	-2,41		
SLK	-3,41	EVI5	-2,41		
gene:ENSSSCG00000036274	-3,41	ANXA13	-2,37		
DLGAP5	-3,40	EIF2A	-2,36		
gene:ENSSSCG00000038557	-3,40	TAX1BP1	-2,36		
ITGB3	-3,40	SRP72	-2,35		
SMARCC2	-3,40	DHX40	-2,35		
HUWE1	-3,39	COPB2	-2,35		
VWA1	-3,39	gene:ENSSSCG00000003903	-2,35		
EFNB1	-3,38	GLUD1	-2,32		

PTPRF	-3,38	FXR1	-2,32		
EPCAM	-3,36	CFDP1	-2,30		
CXCL14	-3,35	NUCKS1	-2,30		
FN1	-3,34	CPD	-2,30		
ACSL4	-3,34	SEPTIN7	-2,29		
SMS	-3,33	CCN3	-2,28		
gene:ENSSSCG00000038144	-3,32	gene:ENSSSCG00000035209	-2,28		
CPNE3	-3,31	gene:ENSSSCG00000016330	-2,26		
DEPDC1B	-3,31	CCDC80	-2,26		
OPTN	-3,27	BHLHE41	-2,26		
CCDC47	-3,25	FLNA	-2,25		
PPP2R3C	-3,25	PNISR	-2,25		
DST	-3,25	RSF1	-2,23		
CDH6	-3,23	SUCLG2	-2,21		
TCEA3	-3,21	gene:ENSSSCG00000000697	-2,21		
PAPSS1	-3,20	gene:ENSSSCG00000033497	-2,17		
ERO1A	-3,20	gene:ENSSSCG00000007322	-2,17		
DSP	-3,18	gene:ENSSSCG00000017164	-2,14		
BAZ1B	-3,17	VIM	-2,13		
AP2M1	-3,16	PTGES	-2,10		
LAMP1	-3,14	EPCAM	-2,10		
CAP1	-3,13	HK1	-2,10		
gene:ENSSSCG00000040337	-3,13	ANKRD12	-2,06		
ADAM9	-3,13	RDX	-2,06		
VPS35	-3,12	LDHA	-2,04		
BCAM	-3,10	RBPJ	-2,03		
DHX40	-3,09	PDIA3	-1,97		
CPD	-3,08				
TUBB2B	-3,07				
RBBP7	-3,06				
RSF1	-3,04				
gene:ENSSSCG00000002825	-3,03				
VIRMA	-3,03				
CETN3	-2,99				
SHTN1	-2,99				
AP2B1	-2,99				
PTGES	-2,98				
VDAC1	-2,96				
CLGN	-2,96				
COPB2	-2,96				
TPP1	-2,96				

CHD9	-2,96				
CMIP	-2,96				
CCDC181	-2,95				
KRT14	-2,94				
PLEC	-2,94				
PDGFA	-2,94				
RAB6A	-2,93				
COPA	-2,93				
TM9SF4	-2,92				
GLG1	-2,90				
gene:ENSSSCG00000016330	-2,90				
ZNF395	-2,90				
CCDC88A	-2,86				
NUCKS1	-2,84				
PRKCSH	-2,83				
RDX	-2,83				
TLN1	-2,83				
F3	-2,82				
FLNA	-2,80				
P4HA2	-2,80				
USP8	-2,80				
FLNB	-2,80				
gene:ENSSSCG00000017783	-2,78				
gene:ENSSSCG00000035209	-2,78				
PMEPA1	-2,76				
EVI5	-2,76				
HADHA	-2,75				
PSME1	-2,74				
BCAT1	-2,71				
TAX1BP1	-2,71				
PHACTR4	-2,70				
TTC3	-2,70				
gene:ENSSSCG00000007314	-2,70				
BAIAP2	-2,69				
HDAC7	-2,69				
CBX5	-2,67				
CENPF	-2,66				
gene:ENSSSCG00000017164	-2,64				
APP	-2,61				
BRD7	-2,61				
SMC3	-2,60				

SUPT3H	-2,58				
RBPJ	-2,57				
FNDC3B	-2,56				
SEPTIN10	-2,55				
TOP2B	-2,55				
H2AC20	-2,54				
gene:ENSSSCG00000022032	-2,54				
AP1B1	-2,54				
LAP3	-2,53				
CST6	-2,52				
GPD2	-2,52				
NAP1L1	-2,50				
FZD5	-2,49				
SUCLG2	-2,49				
P4HB	-2,49				
FZD6	-2,48				
RNF181	-2,48				
gene:ENSSSCG00000048341	-2,47				
TUSC3	-2,47				
WFDC2	-2,47				
NDFIP1	-2,46				
PTPN11	-2,45				
gene:ENSSSCG00000000261	-2,44				
SEC63	-2,43				
UQCRC1	-2,43				
EIF4G3	-2,42				
NANS	-2,42				
HSPD1	-2,42				
DDX6	-2,42				
gene:ENSSSCG00000000697	-2,41				
gene:ENSSSCG00000040827	-2,41				
HSP90B1	-2,41				
PPP1R12A	-2,41				
H1-0	-2,40				
GLUD1	-2,40				
RAB10	-2,39				
SORBS2	-2,38				
SCD	-2,38				
PNISR	-2,37				
BCL2L1	-2,37				
PDLIM7	-2,37				

VIM	-2,37				
MAP4K4	-2,36				
CCDC186	-2,35				
gene:ENSSSCG00000033385	-2,33				
CAST	-2,33				
gene:ENSSSCG00000005518	-2,32				
MEST	-2,32				
ATP5F1A	-2,31				
RNF19A	-2,31				
ANKRD12	-2,30				
DYNC1LI2	-2,30				
LGALS1	-2,30				
ICAM1	-2,28				
CANX	-2,28				
LDHA	-2,28				
CSDE1	-2,28				
SEPTIN7	-2,27				
MXI1	-2,26				
TMEM50A	-2,25				
LY6D	-2,25				
EIF4B	-2,23				
P4HA1	-2,23				
BAZ2A	-2,23				
EEF1G	-2,23				
SMARCE1	-2,22				
CARHSP1	-2,22				
HSP90AB1	-2,21				
gene:ENSSSCG00000038594	-2,20				
gene:ENSSSCG00000049831	-2,18				
MAP1B	-2,18				
NIPBL	-2,18				
ST13	-2,16				
PSIP1	-2,15				
ARPC2	-2,15				
MSN	-2,14				
VPS28	-2,13				
ALDOA	-2,13				
LMO7	-2,12				
METAP2	-2,10				
PARK7	-2,09				
TOP2A	-2,07				

AKAP9	-2,04				
gene:ENSSSCG00000039658	-2,03				
gene:ENSSSCG00000001930	-2,02				
EIF3F	-2,02				
GAPDH	-2,01				
DYNC1I2	-2,01				
S100A4	-1,98				
PPIG	-1,98				
RPL6	-1,93				
RPS6	-1,78				

Fold change of gene expression in infected cells comparing to control (mock-infected cells).
