# Supplementary material for: Porcine Circovirus Modulates Swine Influenza Virus Replication in Pig Tracheal Epithelial Cells and Porcine Alveolar Macrophages
Source: Viruses. 2023 May 20;15(5):1207. doi: 10.3390/v15051207 (PMC10222781; doi:10.3390/v15051207)
Supplement: Supplementary file 1 [file viruses-15-01207-s001.zip › Supplemental Table S4. Up-rgulated genes in iPAM .pdf]

Supplemental Table S4. Up-regulated genes in iPAM 3D4/21 cells

PCV2b/H1N1		PCV2b		H1N1	
Gene ID	Fold change	Gene ID	Fold change	Gene ID	Fold change
FAM124A	246,10	RCSD1	217,99	CCK	181,02
MX2	235,55	FAM124A	200,88	IL19	98,10
ARG1	200,86	ARG1	195,20	RCSD1	21,56
VAT1L	182,81	VAT1L	165,05	gene:ENSSSCG00000043470	18,83
CCK	174,13	SLC38A4	134,88	C3	13,90
SLC38A4	146,43	CCK	122,35	TNFAIP3	9,59
RCSD1	113,02	MX2	110,08	S100A8	9,49
IL19	70,79	IL19	71,13	TNIP3	8,60
CXCL2	35,69	CHRNG	40,13	CXCL2	8,24
ISG15	35,34	ISG15	28,95	NLRP3	8,08
gene:ENSSSCG00000045989	32,50	B3GNT9	26,12	PLET1	6,10
B3GNT9	28,46	gene:ENSSSCG00000049288	23,38	PTGS2	5,96
CHRNG	27,13	CXCL2	22,51	HS3ST1	5,44
gene:ENSSSCG00000022738	20,66	gene:ENSSSCG00000022738	19,10	CD14	4,74
ISG12(A)	19,86	ISG12(A)	18,00	EHF	4,25
gene:ENSSSCG00000032591	17,87	CABP5	17,38	ZC3H12A	4,17
TNFAIP3	17,26	PRDM5	14,94	gene:ENSSSCG00000006588	4,14
PTGS2	17,23	S100A8	13,64	ANKRD1	4,12
TNIP3	15,35	gene:ENSSSCG00000046436	13,33	IL1A	4,05
IQUB	14,65	IQUB	13,08	STYXL1	4,04
IFIT1	14,51	PTGS2	11,41	ZC3H12C	3,99
CABP5	13,45	DHRS9	10,49	ING3	3,66
S100A8	12,59	CCL2	10,48	S100A12	3,55
gene:ENSSSCG00000049288	12,25	NLRP3	10,09	gene:ENSSSCG00000014997	3,54
gene:ENSSSCG00000016733	12,14	TGFBR3	9,79	CHI3L1	3,29
KLF4	11,67	TNFAIP3	9,74	ITPR1	3,24
NLRP3	9,74	ZC3H12A	9,55	ICAM1	3,14
TGFBR3	9,13	gene:ENSSSCG00000032591	9,51	PIGR	3,12
PRDM5	8,96	IFIT1	8,46	F3	2,97
MX1	8,90	PAPPA	7,87	FBXO32	2,94
DHRS9	8,67	gene:ENSSSCG00000047320	7,79	RNF125	2,78
CHAC1	8,61	AGR2	7,74	APIP	2,75
PLET1	8,60	gene:ENSSSCG00000049253	7,63	MX1	2,73
gene:ENSSSCG00000043470	7,98	TNIP3	7,47	gene:ENSSSCG00000020872	2,70
IL1A	7,94	CDC6	7,34	HPS5	2,52

gene:ENSSSCG00000010878	7,52	C3	7,25	TNIP1	2,46
ATP5MC3	7,25	gene:ENSSSCG00000010878	7,11	IER3	2,43
C3	7,21	EDN1	6,92	CD44	2,41
PMAIP1	7,16	MX1	6,30	NFKBIZ	2,37
ATG101	7,02	PSPH	6,23	PLTP	2,34
gene:ENSSSCG00000008929	6,79	MMP13	6,23	TXNRD1	2,32
NUPR1	6,42	gene:ENSSSCG00000017864	6,07	ATF3	2,25
EDN1	6,10	gene:ENSSSCG00000044847	6,07	CFTR	2,18
CDC6	6,09	PLET1	5,92	gene:ENSSSCG00000010954	2,03
gene:ENSSSCG00000047320	6,08	TPST1	5,69	COX1	1,99
SERPINE1	6,05	gene:ENSSSCG00000038304	5,55	TNFRSF1B	1,90
gene:ENSSSCG00000024973	5,82	ND6	5,50	EREG	1,89
TRIB3	5,66	HOOK2	5,45	GSAP	1,84
TPST1	5,63	ATG101	5,36	PADI1	1,84
ANKRD1	5,33	GLP2R	5,22		
OLFML3	5,28	gene:ENSSSCG00000024973	5,19		
RASSF1	5,20	SOX9	5,16		
HS3ST1	5,13	MLLT11	5,11		
IVL	5,12	gene:ENSSSCG00000026210	5,11		
gene:ENSSSCG00000014997	5,06	S100A12	4,91		
IFIT2	5,01	PMAIP1	4,90		
gene:ENSSSCG00000044847	5,01	RASSF1	4,75		
SOX9	4,80	SERPINE1	4,62		
MAP3K8	4,79	MXD1	4,57		
gene:ENSSSCG00000006588	4,73	IVL	4,57		
CHI3L1	4,50	TDG	4,57		
SLC1A4	4,49	RNF19B	4,52		
ZC3H12C	4,39	ANKRD1	4,50		
FOS	4,37	NFKBIA	4,42		
IL6	4,37	RGS2	4,33		
PPP1R15A	4,33	IER3	4,24		
MMP13	4,27	MAP3K8	4,21		
IFIH1	4,18	GMNN	4,18		
gene:ENSSSCG00000012050	4,16	CSKMT	4,09		
IER3	4,09	gene:ENSSSCG00000012050	4,07		
EPSTI1	4,06	GADD45B	3,95		
LIPG	4,03	IL1A	3,94		
IFNGR1	4,01	AGPAT4	3,93		
EHF	3,99	SEMA7A	3,93		
ITPR1	3,98	gene:ENSSSCG00000006588	3,89		
HERC5	3,98	CCDC80	3,83		

GADD45B	3,95	ZNFX1	3,77		
ING3	3,93	SERTAD1	3,70		
F3	3,91	CHI3L1	3,70		
PLAU	3,88	NFKBIZ	3,69		
CCN2	3,86	CCN2	3,67		
NFKBIA	3,80	HBEGF	3,65		
GADD45A	3,74	RND3	3,65		
ZC3H12A	3,68	IFFO2	3,63		
ATF3	3,68	gene:ENSSSCG00000047164	3,59		
S100A12	3,65	TMPRSS11F	3,47		
PSPH	3,62	THOC6	3,45		
gene:ENSSSCG00000013613	3,61	ZC3H12C	3,44		
NRP2	3,50	ICAM1	3,37		
EGR1	3,40	MAFG	3,35		
TTC9	3,32	SAT1	3,29		
FBXO32	3,29	ADM	3,24		
OAS2	3,27	ZNF131	3,24		
BATF3	3,23	F3	3,22		
gene:ENSSSCG00000009633	3,22	IFNGR1	3,18		
VEGFA	3,21	COX1	3,16		
RND3	3,19	HS3ST1	3,15		
ICAM1	3,14	gene:ENSSSCG00000017106	3,13		
CSKMT	3,10	HPS5	3,11		
JMY	3,08	S1PR1	3,08		
EREG	3,06	CUTC	3,08		
TMPRSS11F	3,02	gene:ENSSSCG00000014997	3,04		
HBEGF	2,99	HERC5	3,02		
CTSC	2,97	APOBEC1	2,89		
SGMS2	2,92	RSRP1	2,88		
NMI	2,89	gene:ENSSSCG00000009633	2,86		
APOBEC1	2,87	gene:ENSSSCG00000033189	2,83		
PLK2	2,86	FJX1	2,81		
TMEM87B	2,85	EPSTI1	2,80		
CENPS	2,82	MMP7	2,80		
CD44	2,81	NUPR1	2,76		
CSTF2	2,80	CCNL1	2,76		
H1-3	2,80	DDX52	2,73		
gene:ENSSSCG00000003079	2,73	EREG	2,73		
NFKBIZ	2,69	LIPG	2,62		
COX1	2,69	TRIB1	2,61		
MKNK2	2,67	BRIX1	2,61		

MED13	2,56	C1orf52	2,58		
TNIP1	2,54	MALT1	2,58		
ZNFX1	2,53	SIRT7	2,57		
TARS1	2,50	GADD45A	2,56		
HPS5	2,49	OAS2	2,55		
CDCP1	2,35	EHF	2,55		
YPEL2	2,33	RBP4	2,53		
gene:ENSSSCG00000032525	2,30	FST	2,51		
SAT1	2,21	FAM76B	2,48		
BRIX1	2,20	CBX2	2,44		
SAMD9	2,19	SECISBP2L	2,41		
SERPINE2	2,16	AMOTL2	2,41		
JAG1	2,16	PLK2	2,41		
ATF4	2,05	GID4	2,40		
EIF2AK2	2,04	IFITM3	2,39		
PSME2	2,02	TP53RK	2,39		
EIF4EBP1	1,96	TNFSF9	2,38		
EIF3J	1,94	gene:ENSSSCG00000016441	2,38		
ACSL4	1,85	CTSC	2,37		
PADI1	1,83	TMEM87B	2,37		
		WDR74	2,32		
		CFD	2,31		
		TNIP1	2,31		
		JAG1	2,31		
		CSTF2	2,29		
		FKBP4	2,27		
		ATF3	2,25		
		KLF6	2,23		
		R3HDM4	2,20		
		gene:ENSSSCG00000003079	2,19		
		MGLL	2,17		
		AMD1	2,15		
		MAP2K3	2,11		
		gene:ENSSSCG00000017754	2,08		
		BST2	2,07		
		BMP4	2,07		
		FBXO32	2,06		
		FAM177A1	2,06		
		gene:ENSSSCG00000011803	2,05		
		CCND1	2,04		
		CD44	2,03		

		ZFP36L1	2,01		
		gene:ENSSSCG00000028092	2,00		
		IVNS1ABP	1,97		
		ID2	1,96		
		CLDN1	1,95		
		TRIM25	1,93		
		NAMPT	1,92		
		PLEKHN1	1,92		
		MKNK2	1,91		
		TAF1D	1,89		
		EMP1	1,89		
		TRA2B	1,88		
		FSCN1	1,87		
		DNAJB1	1,86		
		SAMD9	1,84		
		SNAPC1	1,84		
		SERPINE2	1,82		
		EIF3J	1,82		
		TM4SF1	1,79		
		SPP1	1,77		
		POLR3D	1,77		
		SDC1	1,73		
		RSRC2	1,68		
		MAP7D1	1,68		
		SRXN1	1,63		
		LLPH	1,59		

Fold change of gene expression in infected cells comparing to control (mock-infected cells).
