# Supplementary material for: Porcine Circovirus Modulates Swine Influenza Virus Replication in Pig Tracheal Epithelial Cells and Porcine Alveolar Macrophages
Source: Viruses. 2023 May 20;15(5):1207. doi: 10.3390/v15051207 (PMC10222781; doi:10.3390/v15051207)
Supplement: Supplementary file 1 [file viruses-15-01207-s001.zip › Supplemental Table S5. Down-rgulated genes in iPAM.pdf]

Supplemental Table S5. Down-regulated genes in iPAM 3D4/21 cells

PCV2b/H1N1		PCV2b		H1N1	
Gene ID	Fold change	Gene ID	Fold change	Gene ID	Fold change
MGP	-28,88	SENP5	-9,48	MGP	-39,64
GSTA1	-6,75	MGP	-6,54	WNT16	-4,73
MAP2	-5,32	EPS8	-5,77	BMP7	-4,46
NECTIN1	-4,08	TBC1D5	-5,49	FAM110C	-2,60
SEMA3E	-3,94	PCSK5	-5,47	FIBIN	-2,57
PHACTR2	-3,63	FLRT3	-5,30	SCIN	-2,51
MARCKS	-3,25	WDR11	-4,54	IL1RL1	-2,43
SCIN	-3,03	gene:ENSSSCG00000006725	-4,50	ZBTB20	-2,32
DMXL2	-2,95	ATM	-4,25	MARCKS	-2,10
gene:ENSSSCG00000023325	-2,95	SEMA3E	-4,24	SELENOP	-2,07
BICC1	-2,91	PLXDC2	-3,94	COG3	-2,04
HOMER1	-2,69	PBX3	-3,69	TCEA3	-1,98
KRT14	-2,58	USP13	-3,51	GSTA1	-1,94
SELENOP	-2,58	ARHGAP42	-3,44	PTPN13	-1,92
TCEA3	-2,56	gene:ENSSSCG00000032297	-3,41	C1orf21	-1,76
LAMP1	-2,54	EXOC6B	-3,34		
FARP1	-2,46	PGAP1	-3,32		
WWC1	-2,43	GSTA1	-3,28		
ARHGDIB	-2,29	TUT4	-3,26		
ADAMTS1	-2,27	PPP2R2B	-3,26		
PPP2R5A	-2,25	ASXL2	-3,21		
PLS1	-2,25	MGAT4C	-3,17		
SRPK2	-2,22	MAP2	-3,15		
SMARCA2	-2,13	AGL	-3,13		
PTPN13	-2,03	FAM53B	-3,03		
MET	-2,01	CERT1	-3,01		
TPP1	-2,00	GTF2I	-2,98		
		PCLO	-2,91		
		FAM110C	-2,89		
		HOMER1	-2,89		
		BICC1	-2,86		
		DIP2B	-2,80		
		DMXL2	-2,76		

		WWC1	-2,71		
		PSME3IP1	-2,69		
		TCEA3	-2,69		
		SMARCC2	-2,67		
		NECTIN1	-2,67		
		SLC16A7	-2,66		
		PRKD3	-2,65		
		URI1	-2,65		
		DCDC2	-2,62		
		KIAA1217	-2,61		
		PIBF1	-2,60		
		NRDC	-2,59		
		CASK	-2,58		
		ARL13B	-2,57		
		MGME1	-2,56		
		PTPN13	-2,51		
		PLS1	-2,51		
		gene:ENSSSCG00000010401	-2,50		
		SLC37A4	-2,49		
		IQGAP2	-2,49		
		SREBF2	-2,46		
		ACAP2	-2,46		
		CNKSR2	-2,46		
		SMARCA2	-2,45		
		ZNF644	-2,43		
		LAMP1	-2,43		
		LAMC1	-2,42		
		gene:ENSSSCG00000023325	-2,36		
		R3HDM2	-2,32		
		ARID5B	-2,31		
		SF3A3	-2,28		
		CLCN5	-2,26		
		MAL	-2,26		
		MARCKS	-2,24		
		PKD2	-2,24		
		NDUFV1	-2,23		
		MACC1	-2,22		
		USP47	-2,21		
		NR3C1	-2,19		
		ASPM	-2,19		
		gene:ENSSSCG00000009501	-2,17		

		FARP1	-2,16		
		PALMD	-2,16		
		EML4	-2,15		
		ZMYND11	-2,13		
		ENPP4	-2,11		
		PTPRZ1	-2,11		
		GNS	-2,10		
		ACSL3	-2,08		
		MET	-2,08		
		ARHGEF12	-2,07		
		SELENOP	-2,07		
		AP1B1	-2,07		
		SORT1	-2,06		
		SORBS1	-2,06		
		IGF2BP3	-2,05		
		GLG1	-2,05		
		PPP1R21	-2,05		
		NPNT	-2,05		
		UBR5	-2,01		
		GOLIM4	-2,00		
		AIFM1	-1,99		
		ADD3	-1,99		
		AP3D1	-1,98		
		TEAD1	-1,97		
		gene:ENSSSCG00000017783	-1,97		
		PPP2R5A	-1,96		
		LAMB1	-1,96		
		TTC3	-1,95		
		APLP2	-1,94		
		ITCH	-1,93		
		NECTIN3	-1,93		
		CRIM1	-1,92		
		DDX46	-1,92		
		ECPAS	-1,88		
		CXADR	-1,88		
		RB1CC1	-1,86		
		ATP11C	-1,86		
		FOXN3	-1,84		
		ATP6V1C1	-1,84		
		SLC39A10	-1,82		
		gene:ENSSSCG00000002722	-1,82		

		IRF2BP2	-1,82		
		SYNE2	-1,81		
		SPTBN1	-1,80		
		KRT14	-1,80		
		HNRNPR	-1,80		
		IARS1	-1,79		
		gene:ENSSSCG00000012102	-1,77		
		EHHADH	-1,77		
		RSRC1	-1,75		
		ATRX	-1,71		
		NASP	-1,69		
		MYO6	-1,67		
		PSMD2	-1,66		
		PTGFRN	-1,66		
		CALD1	-1,66		
		SMARCC1	-1,65		
		TOP2B	-1,62		

Fold change of gene expression in infected cells comparing to control (mock-infected cells).
